# Anti-inflammatory and Antioxidant Activity of Cucumis sativus and Citrus macroptera Herbal Formulation: An In-Vitro Study

**DOI:** 10.7759/cureus.51818

**Published:** 2024-01-07

**Authors:** Turaga Amani, Mouttoukichenin Surenthar, Rajeshkumar Shanmugam

**Affiliations:** 1 Oral Medicine and Radiology, Saveetha Dental College and Hospitals, Saveetha Institute of Medical and Technical Sciences, Saveetha University, Chennai, IND; 2 Pharmacology, Saveetha Dental College and Hospitals, Saveetha Institute of Medical and Technical Sciences, Saveetha University, Chennai, IND

**Keywords:** lemon, cucumber, citrus macroptera, cucumis sativus, anti-inflammatory activity, antioxidant activity, dental, innovation and health

## Abstract

Background

The interest in natural remedies is increasing rapidly as they can serve as alternatives to synthetic drugs and reduce their potential side effects. Current research is focused on examining the antioxidant and anti-inflammatory characteristics of a combination of *Cucumis sativus* and C*itrus macroptera* extract in an in-vitro context. The combination of citrus, cucumber, and glycerol extract could serve as an effective alternative to synthetic antioxidants and anti-inflammatory drugs and lower the side effects of the available synthetic drugs. This extract can be used to treat potentially malignant oral disorders like oral leukoplakia, oral submucous fibrosis, and oral lichen planus, which are attributed to its antioxidant properties.

Aim

To evaluate the anti-inflammatory and antioxidant activity of formulation of *C. sativus*, *C. macroptera*, and glycerol extract.

Materials and methods

The cucumber and citrus fruits were separated, cleaned, and dried. The cucumber was then peeled, its seeds cleaned, and the pulp cut into pieces. Using a laboratory mortar and pestle, a 5 ml extract was prepared. The citrus fruit was cut in half, its seeds were removed, and a 5 ml extract was prepared from their pulp only. These two extracts were combined to form a 10 ml extract solution with 8 ml of glycerol. The extracts were combined, shaken for 24 hours, filtered, and stored at 4°C. Assays like the 2,2-diphenyl-1-picrylhydrazyl (DPPH) radical scavenging assay, Hydrogen Peroxide (H2O2) assay, Bovine serum albumin (BSA) assay, and Egg albumin (EA) denaturation assay were performed to assess their anti-inflammatory and antioxidant properties.

Results

The antioxidant and anti-inflammatory properties of the extract showed comparable activity (percentage of inhibition: 76% in BSA and EA assays; 90% in DPPH and H2O2 assays) to that of the standard values (percentage of inhibition: 78% in BSA and EA assays; 92% in DPPH and H2O2 assays) at concentrations 30, 40, and 50 µl in the BSA assay, EA assay, DPPH, and H2O2 assay. The maximum concentration at which the antioxidant and anti-inflammatory effects were appreciable was 50 µl in all assays.

Conclusion

This study concluded that the combination of cucumber, citrus, and glycerol extract could serve as an effective alternative to synthetic antioxidants and anti-inflammatory drugs currently available. These extracts can provide a promising solution in the field of drug development for treating lesions caused by free radicals and oxidative stress in the oral cavity.

## Introduction

A member of the Cucurbitaceae family named Cucumis sativus L. is widely recognized as a Cucumber in English, Khira in Hindi, and Sakusa in Sanskrit. It is naturally found in the Himalayan regions and extensively cultivated across India. This plant's fruit juice has been used as food, and its seeds have been used for their cooling and diuretic effects to treat headaches in traditional practices [1,2]. The Citrus genus encompasses numerous economically significant fruits like Citrus medica, Citrus aurantifolia, Citrus sinensis, and Citrus reticulata, which are cultivated globally due to their rich nutritional and medicinal benefits [3]. Citrus belongs to the Rutaceae family, among the largest families within the Sapindales order. Typically, citrus flowers and leaves have strong scents, and their extracts are rich in important flavonoids and other substances that work well as insecticides, fungicides, and therapeutic agents. Citrus macroptera is a semi-wild Citrus species indigenous to Malesia and Melanesia regions. This Citrus macroptera plant is cultivated in many homesteads and hilly areas within the Sylhet division of Bangladesh [4]. In India, it has become established in the northeastern region. Studies have indicated that C. macroptera fruits possess anti-diabetic properties when tested in experimental type 2 diabetic rats [5]. Additionally, the fruit peel of C. macroptera has demonstrated notable neuropharmacological effects in combating oxidative stress [6].

Cancer is a medical condition characterized by uncontrolled cell growth, and its treatment typically involves various methods, including radiotherapy, chemotherapy, and complex surgical procedures. Conventional chemotherapy entails the indiscriminate administration of drugs via intravenous routes, often leading to significant adverse effects on healthy adjacent tissues, thereby limiting its efficacy [7]. Chemotherapy is linked to a range of negative outcomes, including hair loss, weight loss, nausea, skin issues, diarrhea, and insomnia. Additionally, the poor solubility of hydrophobic drugs adversely affects their absorption and pharmacokinetics. This challenges the quest for new therapeutic agents that offer reduced side effects and fewer limitations. Anticancer treatments have made significant advancements with the introduction of nanotechnology [8]. The antioxidant properties of nanotechnology arise from its ability to scavenge reactive oxygen species (ROS), thereby mitigating their harmful effects [9].

Acute inflammation is brought on by alterations in the blood arteries near the wounded area, which become more permeable and permit the release of plasma proteins and white blood cells into the surrounding tissue. As a result of the increased flow of fluids into the tissue, inflammation-related edema develops [10]. Various well-known anti-inflammatory mechanisms also follow, such as suppressing the expression of pro-inflammatory cytokine genes, modifying the production of pro-inflammatory cytokine proteins, reducing the activity of the nuclear factor kappa B (NF-κB) pathway, selectively targeting the enzyme activity of cyclooxygenase (COX-2), inhibiting mast cell degranulation, and down-regulating the release of nitric oxide (NO) by inhibiting the expression of inducible nitric oxide synthase (iNOS) enzyme [11].

Previous studies suggest that Cucumis sativus and Citrus macroptera have antioxidant and anti-inflammatory properties [4,6]. However, the combined effects proposed in this research were not studied previously, which brought novelty to this study. As a result, this study aimed to explore the effects mentioned above to fully benefit from them in oral mucosal lesions, including recurrent aphthous ulcers, potentially malignant disorders like oral leukoplakia, oral lichen planus, and oral submucous fibrosis where inflammations and oxidative stress play a crucial role. The rationale was that using natural remedies as part of a holistic approach to health is beneficial since they not only treat symptoms but also work to improve overall well-being. Natural remedies can be a cure-all for traditional healing. In some cases, individuals can use it in conjunction with allopathic medicines to enhance the overall treatment effect. 

The study aimed to evaluate the antioxidant and anti-inflammatory properties of C. sativus, C. macroptera, and glycerol extract.

## Materials and methods

**Figure 1:**
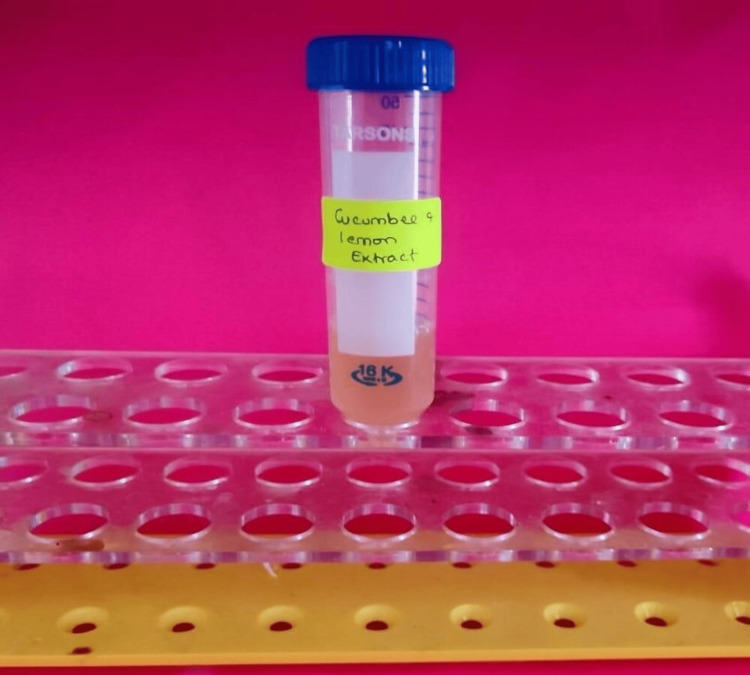
Mixture containing 5ml of cucumber and 5ml of citrus extracts

**Figure 2:**
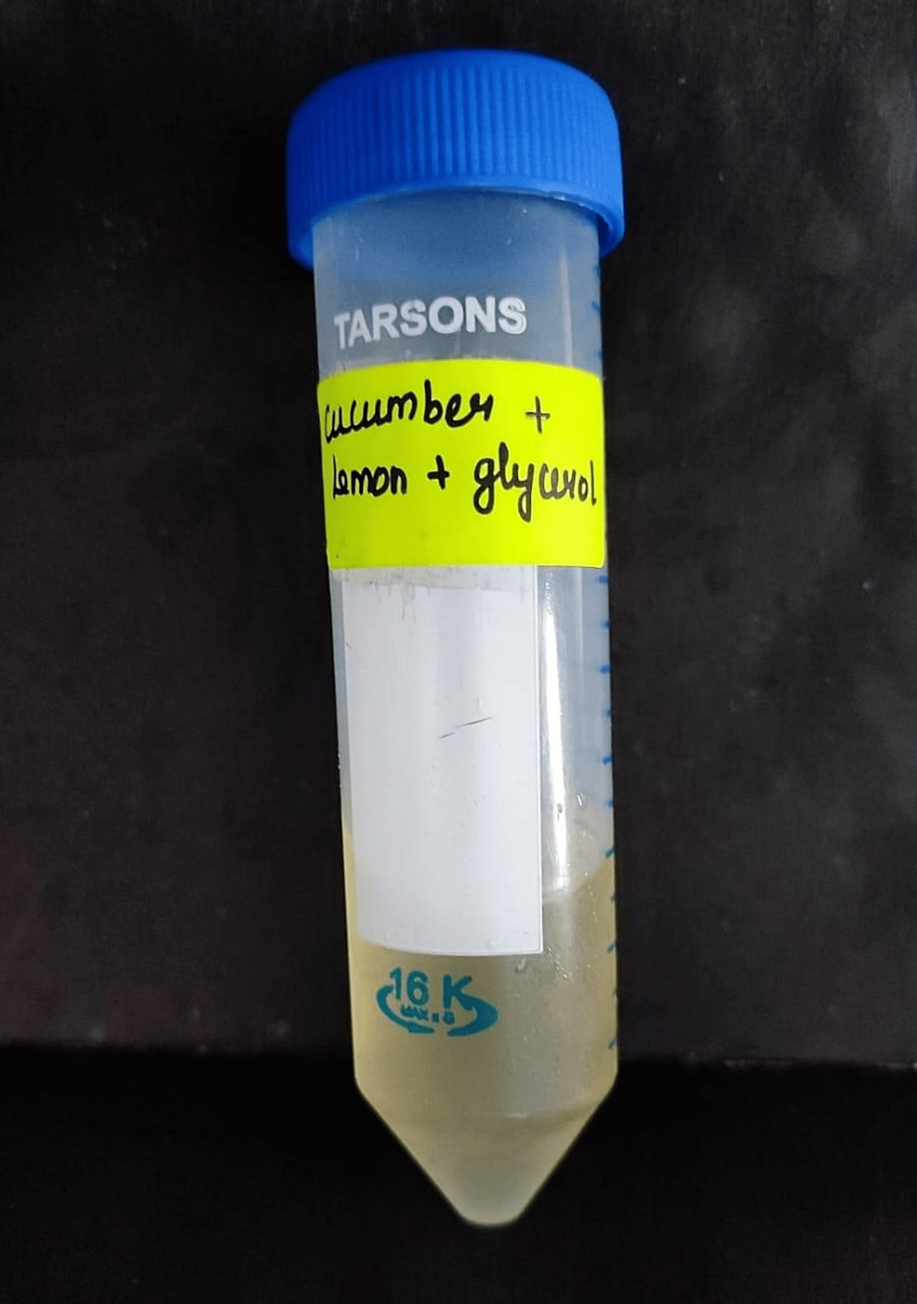
Final extract containing cucumber, citrus, and glycerol

The materials used for this study were Cucumis sativus (cucumber) and Citrus macroptera (lemon), obtained from a local vegetable market. The fruits of citrus were collected in their fully mature form. The extracts were subjected to antioxidant and anti-inflammatory testing. Before the procedure, the cucumber and citrus fruits were separated, cleaned, and dried. The cucumber was then peeled, its seeds cleaned, and the pulp cut into pieces. Using a laboratory motor and pestle, a 5ml extract was prepared. The citrus fruit was cut in half, its seeds were removed, and a 5ml extract was prepared using the pulp only. These two extracts were then combined to form a 10ml extract solution (figure 1) and were combined with 8 ml of glycerol (figure 2).

Cotton plugs were placed on top of the conical flasks containing the extract to prevent evaporation. The extract was shaken for 24 hours at 250 rpm in an orbital shaker. After being shaken overnight, the extract was filtered twice - once with muslin cloth and once with filter paper. The resulting extracts were stored at 4°C. The prepared extract was tested for anti-inflammatory activity using the Bovine serum albumin denaturation (BSA) assay and Egg albumin denaturation (EA) assay.

A. Bovine serum albumin denaturation assay

A total of 0.45 ml of bovine serum albumin was combined with 0.05 ml of cucumber and citrus extracts at varying concentrations ranging from 10 to 50 µg/ml. After adjusting the pH to 6.3, the mixture was incubated for 30 minutes in a water bath heated to 55°C after being kept at room temperature for 10 minutes. The standard reference was diclofenac sodium, while the control was dimethyl sulfoxide. The samples were then put through a spectrophotometric examination using a 660 nm wavelength [12]. The following formula was used to calculate the percentage of protein denaturation:

% inhibition = Absorbance of control - Absorbance of sample/Absorbance of control x 100

B. Egg albumin denaturation assay

A test called the egg albumin denaturation assay was conducted using 0.2 mL of fresh egg albumin mixed with 2.8 ml of phosphate buffer. Different concentrations (ranging from 10-50 µg/ml) of cucumber and lemon extract (5 ml) were added to the mixture. The pH was adjusted to 6.3, and the mixture was kept at room temperature for 10 minutes before being incubated in a water bath at 55°C for 30 minutes. The standard group was diclofenac sodium, while the control group was dimethyl sulfoxide [13]. The samples were then measured spectrophotometrically at 660 nm. The percentage of protein denaturation was calculated using the following equation:

% inhibition = Absorbance of control - Absorbance of sample/Absorbance of control x 100

The prepared extract's antioxidant activity was evaluated through two assays: the 2,2-diphenyl-1-picrylhydrazyl (DPPH) radical scavenging assay and the Hydrogen peroxide assay (H_2_O_2_).

DPPH radical scavenging assay

For this assay, a stock solution of 0.1 mM 2,2-diphenyl-1-picrylhydrazyl (DPPH) was initially prepared using methanol. A fresh working solution was then created by diluting the stock solution with methanol to achieve a final concentration of 20 µM for each assay.

In a 96-well plate, 200 µl of the DPPH working solution was mixed with cucumber and citrus extracts at various concentrations (10, 20, 30, 40, and 50 µg/ml). The dish was then let to sit at room temperature in the dark for 30 minutes. Using a microplate reader and methanol as the blank control, the absorbance was determined at 517 nm after incubation [14]. This formula was used to determine the proportion of DPPH scavenging activity:

% DPPH Scavenging Activity = [(A control - A sample) / A control] × 100

where A control represents the absorbance of the control (DPPH solution without the sample), and A sample is the absorbance of the sample (DPPH solution with the silver nanoparticles synthesized using the green method). The positive control group was treated with ascorbic acid (1 mg/ml).

H_2_O_2_ assay

The hydroxyl radical scavenging assay was used to assess the antioxidant activity of cucumber and citrus extract, as per the method proposed by Halliwell et al. A reaction mixture containing 1 ml of solution and 100 µl of 28mM 2-deoxy-2-ribose was prepared. Then various concentrations (10-50 µg/ml) of cucumber and citrus extract were added, followed by 200 µl of 200 µm ferric chloride, 200 µl of EDTA (ethylenediamine tetraacetic acid), and 100 µl of ascorbic acid respectively. The mixture was incubated for 1 hour at 37°C, and the optical density was measured at 532 nm against the blank solution. As a positive control, Vitamin E was used [15].

Hydroxyl radical scavenging activity (%) = [(A blank - A sample)/A blank] × 100

Where A blank is the absorbance of the control reaction (without sample), and A sample is the absorbance of the reaction with the sample.

## Results

**Figure 3:**
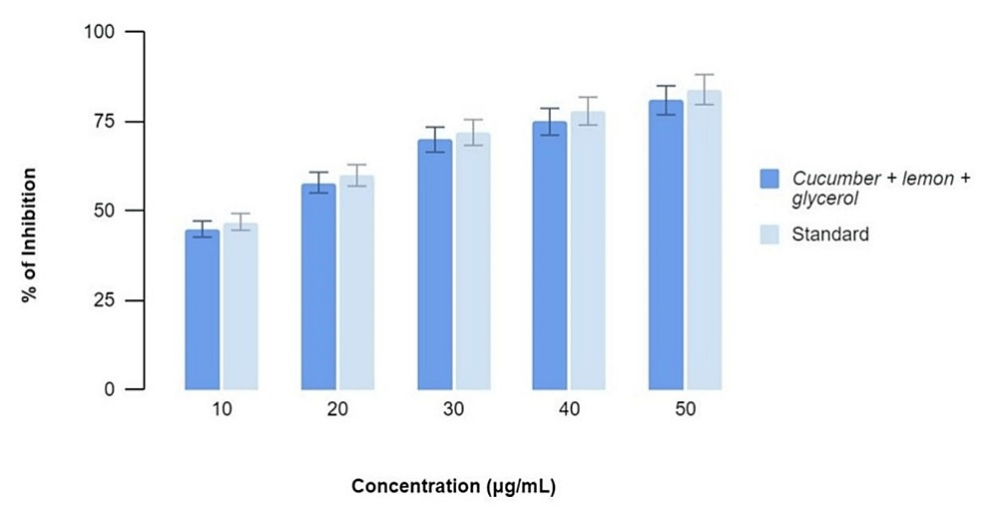
BSA Assay graph demonstrating the percentage of inhibition at various concentrations with equivalent anti-inflammatory activity when the extract and standards are compared BSA: Bovine serum albumin

**Figure 4:**
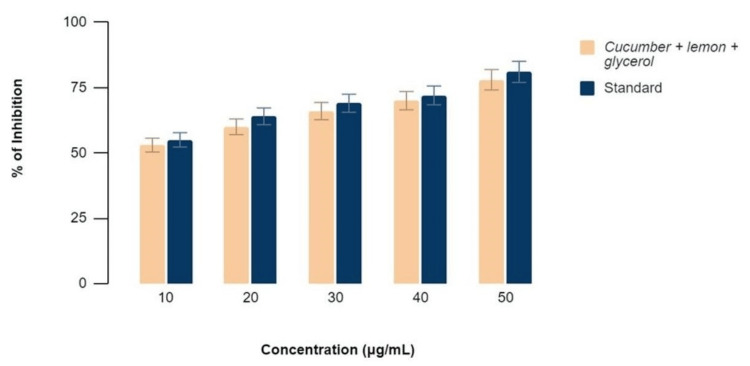
EA Assay demonstrating the percentage of inhibition at various concentrations with similar anti-inflammatory activity when the extract and standards are compared

Figure 3-4 show the anti-inflammatory effects of a mixture of C. sativus, C. macroptera, and glycerol extract using BSA Assay and EA Assay, respectively, compared to standard values at various concentrations. It was found that the values of anti-inflammatory properties of the extract showed comparable activity to that of the standard values at concentrations of 30, 40, and 50 µl. The percentage of inhibition was 48, 54, 72, 74, and 76 at concentrations of 10, 20, 30, 40, and 50 micrograms/ml for both BSA and EA assays.

**Figure 5:**
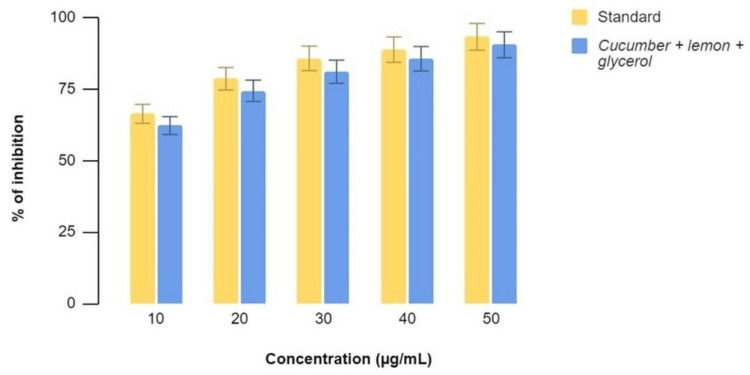
DPPH Assay demonstrating the percentage of inhibition at various concentrations with equivalent antioxidant activity when the extract and standards are compared DPPH: 2,2-diphenyl-1-picrylhydrazyl

**Figure 6:**
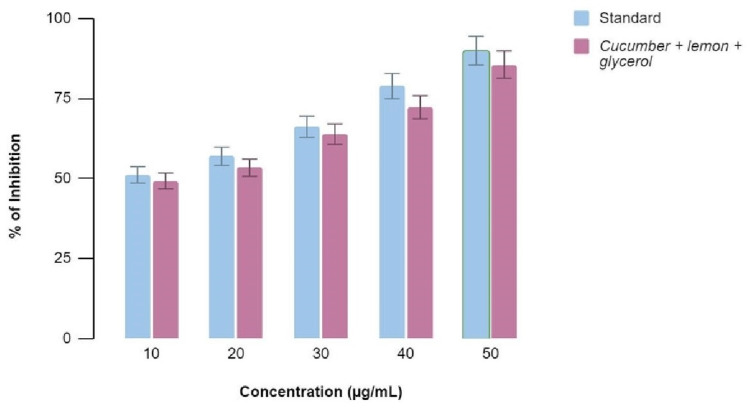
H2O2 Assay demonstrating the percentage of inhibition at various concentrations with similar antioxidant activity when the extract and standards are compared

Figures 5-6 show the antioxidant properties of the combination of C. sativus, C. macroptera, and glycerol extract using DPPH Assay and H_2_O_2_ Assay, respectively, compared to the standard values at various concentrations. It was found that the values of antioxidant properties of the extract showed comparable activity to that of the standard values at concentrations 30, 40, and 50 µl. The percentage of inhibition is 73, 74.5, 81.5, 87.3, and 90 at concentrations of 10, 20, 30, 40, and 50 micrograms/ml for both DPPH and H_2_O_2_ assays.

## Discussion

India is abundant with medicinal plants provided by nature. Herbal extracts are known to possess antioxidant compounds [16]. The study aimed to evaluate the anti-inflammatory and antioxidant activity of the formulation of Cucumis sativus (5 ml), Citrus macroptera (5 ml), and glycerol extract using an in-vitro study. The innovative herbal composition employed in the study, which increased the benefits of utilizing multiple herbs separately by combining them, was its key strength. To the best of our knowledge, the herbal combination used in this study is the first of its type.

Although significant differences in antioxidant and anti-inflammatory parameters were previously observed with these herbs individually, no previous studies were comparing this novel combination. The anti-inflammatory action results from its selective inhibition of prostaglandin E2 synthesis, thromboxane, and other inflammatory mediators of arachidonic acid metabolism [17-19].

Cucumis sativus L., a widely recognized vegetable originally hailing from India, is now commercially grown globally. Additionally, traditional Ayurvedic medicine has long valued its fruits and seeds' cooling and hemostatic tonic properties. Today, it is clear that these fruits harbor a variety of intriguing phytocompounds, making them a compelling choice for antioxidant and anti-inflammatory applications. Cucumis sativus L. extract demonstrated the inhibition of cellular toxicity induced by lipopolysaccharide (LPS) and effectively reduced inflammation-triggered angiogenesis. These compelling and strong findings suggest that Cucumis sativus L. extract holds promise as a natural compound for safeguarding vascular endothelium [20,21,22].

Citrus macroptera Montr., specifically essential oil-derived extracts, possess anti-inflammatory properties and demonstrate notable effectiveness compared to conventional diclofenac sodium. It is widely recognized that protein denaturation is a well-documented contributor to inflammation [23]. The anti-inflammatory tests of the herbal formulation, such as the egg albumin denaturation assay and the bovine serum albumin denaturation assay, revealed that the percentage of inhibition was nearly identical to that of the conventional diclofenac sodium. There was a linear relationship between the percentage of inhibition and extract concentration. This implies that the rise in inhibition percentage is dose-dependent.

Antioxidants are compounds capable of preventing or slowing down cellular damage caused by free radicals within the body. These free radicals are known contributors to the development of various diseases, including many oral potentially malignant disorders, habit-related and non-habit-related oral cancers, and the aging process. The body's metabolic processes can naturally produce antioxidants, referred to as endogenous antioxidants, or acquired from external sources, known as exogenous antioxidants [24].

In a previous study [25], the antioxidant potential of cucumber pulp water extract was evaluated using the DPPH assay, with butylated hydroxytoluene serving as the standard for measuring antioxidant activity. The results indicated that cucumber fruit exhibited substantial antioxidant activity, with an IC50 value (half-maximal inhibitory concentration, the indicator of a drug's effectiveness) of 14.73 ± 1.42 μg/ml.

In another study, the antioxidant activity index (AAI) of different extracts from cucumber pulp and leaves was measured using two methods, DPPH and CUPRAC (CUPric Reducing Antioxidant Capacity). The AAI of DPPH varied from 0.22 to 2.18, while the AAI of CUPRAC ranged from 0.07 to 0.95. The results between the two methods were not linear due to the presence of flavonoids in cucumber pulp extract, which contributed to the antioxidant activity measured by the CUPRAC method. As determined by the DPPH technique, the antioxidant activity was influenced by phenolic components found in the cucumber pulp extract [26]. However, both studies [25,26] suggest that cucumber pulp extract has good antioxidant activity.

Citrus macroptera stands out as a prospective reservoir of natural antioxidants, evident from its elevated levels of polyphenols, flavonoids, tannins, and proteins, along with its notable ability to scavenge DPPH free radicals and substantial FRAP (Ferric Reducing Antioxidant Power) value. Consumption of C. macroptera fruit does not negatively impact vital organ structures or serum biochemical markers, according to an in-vivo examination [27]. Hence, the utilization of this fruit emerges as a promising option for accessing natural antioxidants.

The DPPH radical scavenging assay and H2O2 assay results of the antioxidant tests of the herbal formulation revealed that the percentage of inhibition was nearly equivalent to that of the standard.

Limitation

The research is restricted to in vitro settings, which might not accurately mimic the intricate relationships that take place in the human body. In-vivo studies are required to verify the observed effects in a more comprehensive biological context. Further, variabilities in the content of plant extracts can be introduced by harvesting techniques, climate, and geographic location, which could compromise the repeatability of findings. It is also possible to overlook the synergistic effects of several chemicals in the entire plant or extracts. These drawbacks justify expanding the research's scope in the future.

The future scope is to compare the antioxidant properties of this formulation with the antioxidant properties of lycopene. Further, in-vivo studies and clinical trials can be conducted to determine the clinical efficacy of the cucumber, citrus, and glycerol formulation.

## Conclusions

In conclusion, the study inferred that a combined extract of cucumber, citrus, and glycerol has the potential to serve as effective alternatives to synthetic anti-inflammatory drugs currently available. They can be employed as adjuncts to synthetic anti-inflammatory medications, potentially mitigating drug resistance and reducing overall costs while minimizing side effects. The comparison between the herbal formulation and the standard exhibited a dose-dependent effect. Further research is required to determine the clinical effectiveness in the field of drug development in treating lesions caused by free radicals and oxidative stress in the oral cavity.
